# Clinical update on the hybrid comprehensive stage II operation

**DOI:** 10.1016/j.xjon.2021.04.019

**Published:** 2021-05-08

**Authors:** Michael Farias, Craig E. Fleishman, David Nykanen, William M. DeCampli

**Affiliations:** The Heart Center, Arnold Palmer Hospital for Children Orlando, Fla

**Keywords:** single ventricle, congenital heart disease, hybrid palliation, BDG, bidirectional Glenn, CPB, cardiopulmonary bypass, DOL, day of life, HCS2, hybrid comprehensive stage II, PA, pulmonary artery, PG, Palmaz Genesis, POD, postoperative day, SV, single ventricle, VSD, ventricular septal defect

## Abstract

**Objective:**

We previously described the hybrid comprehensive stage II operation as an alternate surgical procedure for a subset of patients with single ventricle congenital heart disease with adequate native ascending aortic outflow. Here we provide a clinical update on the 4 patients who have undergone this procedure.

**Methods:**

After undergoing a hybrid approach to the stage I Norwood palliation, the hybrid comprehensive stage II procedure was performed with an incision to the main pulmonary artery (PA), dilation of the ductal stent, creation of a stented baffle between the branch PAs, and a bidirectional Glenn connection. With this approach, dissection of the distal arch and creation of a Damus-Kaye-Stansel anastomosis was avoided. A standard Fontan procedure was planned after the usual period of growth.

**Results:**

The first patient, who had trisomy 21 and elevated PA pressures, died postoperatively due to left PA thrombosis. The subsequent 3 patients survived the procedure and remain clinically well. All have required catheterizations for reintervention on their stented intrapulmonary baffles and ductal arches, and all have undergone successful completion of their Fontan procedures.

**Conclusions:**

The hybrid comprehensive stage II is a feasible, less complex alternative to the conventional comprehensive stage II operation in a subset of patients with single ventricle physiology. Early postoperative anticoagulation therapy to avoid PA thrombosis is recommended, and restenting of the ductal arch is anticipated. Although the long-term consequences of separate outflow tracts supplying the upper and lower body is unknown, the 3 surviving patients with this circulation are doing well with their Fontan circulation at midterm follow-up.

**Figure:**
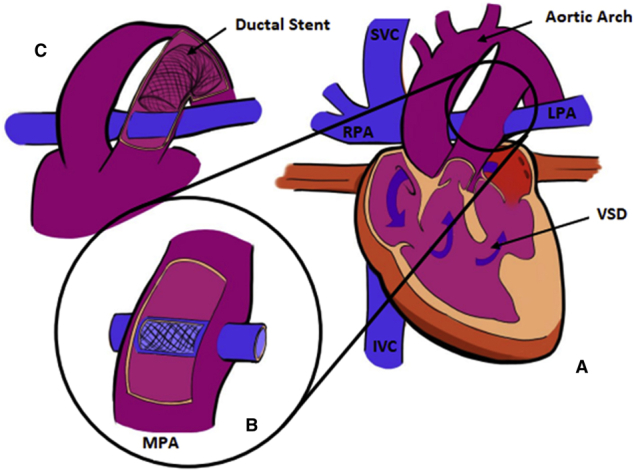
Depictions of completed HCS2 (A) with stented pulmonary baffle (B) and ductal stent (C).

**Central Message**

A hybrid second-stage for single ventricle hearts with adequate aortic flow is a simpler alternative to the comprehensive stage II.

**Perspective**

The hybrid comprehensive stage II operation provides a simpler alternative to the conventional approach in a subset of patients with single ventricle heart disease and adequate ascending aorta outflow. Patency of the ductal arch is maintained and continuity of the pulmonary arteries is created through a stented baffle. Repeat interventions are anticipated on the pulmonary artery baffle and ductal arch.

See Commentary on page 336.

The hybrid Norwood stage I procedure was proposed as an alternative approach to the conventional Norwood stage I operation in patients with hypoplastic left heart syndrome.^1^, ^2^, ^3^ In the hybrid approach, stable circulation is obtained via stenting of the ductal arch to maintain its patency and banding of the branch pulmonary arteries (PAs) to control pulmonary blood flow, with an atrial septostomy performed as needed. The hybrid procedure can be applied to most single ventricle (SV) physiology candidates for the conventional stage I procedure. In some centers it is used routinely, whereas in others it is reserved for higher-risk patients because it offers the advantage of avoidance of neonatal cardiopulmonary bypass (CPB) and possibly deep hypothermia and circulatory arrest. There is high variability in utilization of and outcomes from the hybrid procedure related to both institutional experience and patient selection.^4^, ^5^, ^6^ Furthermore, the purported advantages of avoiding CPB, hypothermia, and selective management of brain circulation are offset by the need for these during the conventional comprehensive stage II operation. During this procedure, in addition to creation of the bidirectional Glenn (BDG) anastomosis, hybrid patients typically require arch reconstruction and creation of the Damus-Kaye-Stansel anastomosis in the presence of a healed-in ductal stent, as well as reconstruction of the previously banded PAs. Although some data suggest improved short-term outcomes for patients undergoing a hybrid approach,^7^ a long-term advantage of this approach over the standard Norwood palliation in terms of mortality and neurodevelopmental outcomes has not been demonstrated.^8^, ^9^, ^10^, ^11^

We previously described the hybrid comprehensive stage II operation (HCS2) as an alternate surgical procedure for a subset of patients with SV physiology with native ascending aortic outflow sufficient for coronary and upper body circulation who have previously undergone the hybrid stage I operation.^12^ This novel procedure reduces the complexity of the stage II operation and therefore sustains much of the purported advantages of the hybrid approach. Here we present a clinical update on 4 patients (Table 1) who have undergone this procedure at our institution, including review of 1 patient (previously reported) with early postoperative death. This case series was reviewed by our institutional review board and a waiver of consent was provided.

**Table 1:** Characteristics of patients undergoing hybrid comprehensive stage II (HCS 2) procedure

Case	Diagnoses	Age at HCS2 (mo)	Age at Fontan (mo)	PA stents required	DA stents required	Age at last follow-up	Status at last follow-up
1	- Trisomy 21 - Unbalanced, right dominant atrioventricular canal - Hypoplastic aortic arch and isthmus	6	N/A	1	1	7 mo	Died POD 26
2	- DORV with mitral atresia and ventricular septal defect - Hypoplastic aortic arch and isthmus	6	34	1	4	~6 y	Alive and well, sats high-90%
3	- DORV with parachute and hypoplastic mitral valve - Hypoplastic aortic arch - Left superior vena cava to coronary sinus	5	37	3	2	4 y	Alive and well, sats high-90%
4	- Mitral atresia with ventricular septal defect - Hypoplastic aortic arch and isthmus - Accessory tissue crowding left ventricular outflow tract	9	36	1	2	~3.5 y	Alive and well, sats low-90%

*HCS 2*, Hybrid comprehensive stage II; *PA*, pulmonary artery; *DA*, ductus arteriosus; *N/A*, not available; *POD*, postoperative day; *DORV*, double-outlet right ventricle; *sats*, oxygen saturation.

## Methods

**Figure 1:**
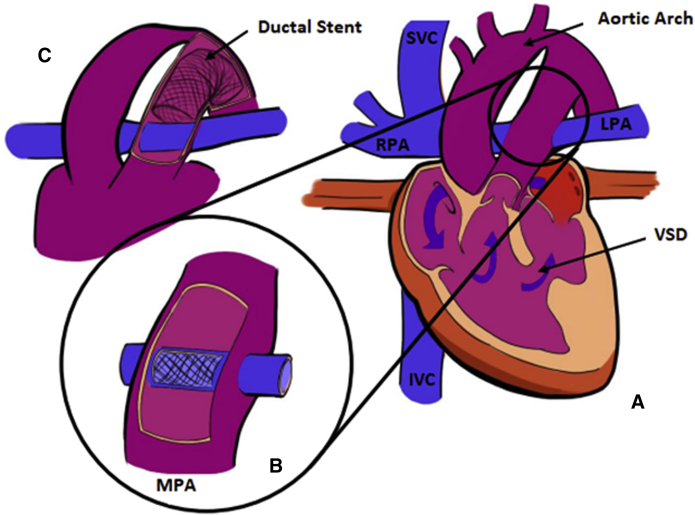
A, Schematic representation of a single ventricle heart after hybrid comprehensive stage II procedure with adequate aortic outflow across a ventricular septal defect (*VSD*) and into the native ascending aorta. B, The pulmonary artery baffle, consisting of a stent covered by a homograft hood, resides within the posterior native main pulmonary artery (*MPA*). C, A stent also resides within the native ductal arch to maintain patency; note that the coarctation in the native aortic arch persists. *SVC*, Superior vena cava; *RPA*, right pulmonary artery; *LPA*, left pulmonary artery; *IVC*, inferior vena cava.

**Figure 2:**
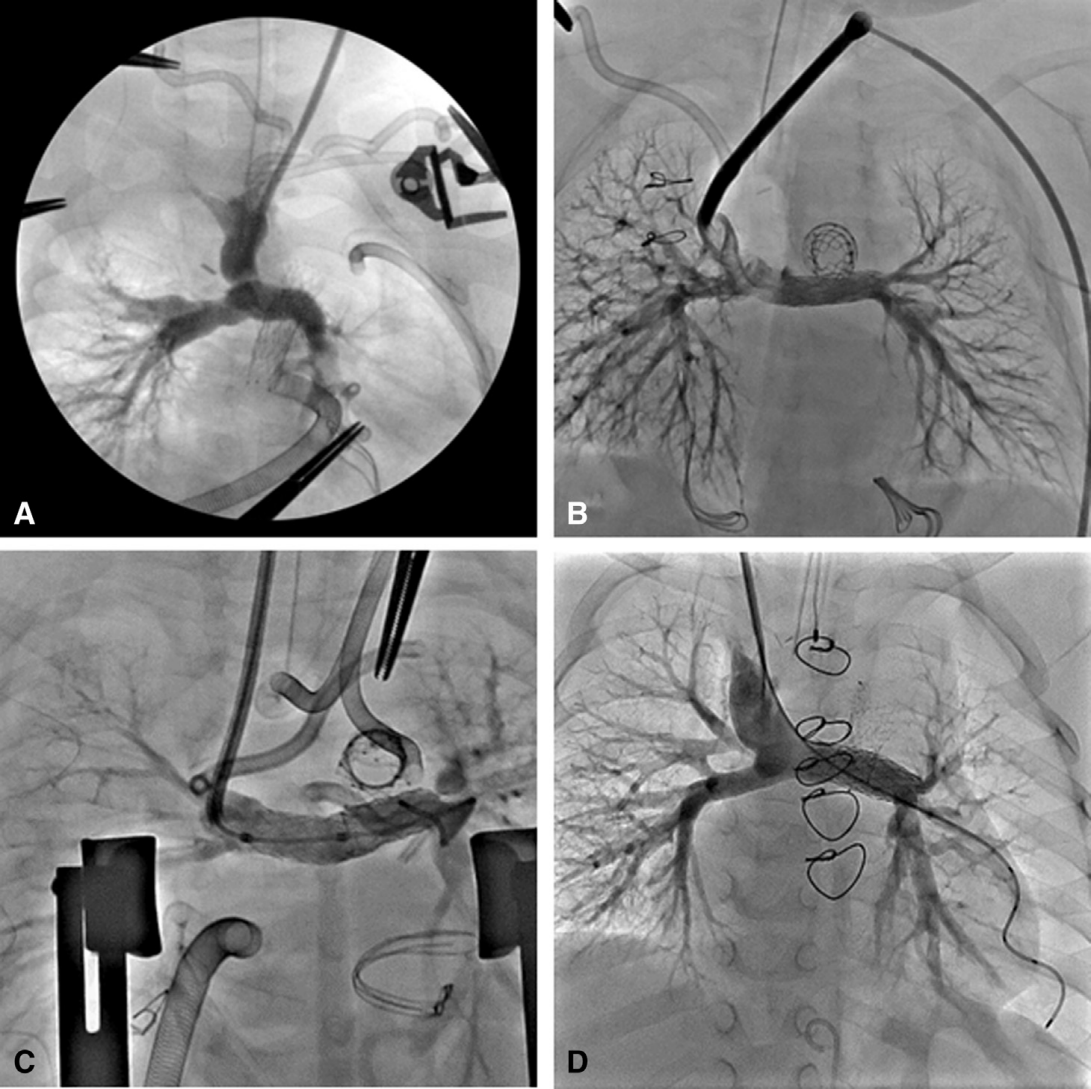
Contrast angiography into the superior vena cava after the bidirectional Glenn procedure demonstrates the patency of the stented pulmonary artery baffle with preserved flow to the left pulmonary artery. A, Contrast angiography of patient 1 taken intraoperatively. B, Contrast angiography of patient 2 taken intraoperatively. C, Contrast angiography of patient 1 taken intraoperatively. D. Contrast angiography of patient 4 taken postoperatively.

Details on the surgical and interventional techniques employed in the HCS2 operation were previously reported.^12^ Briefly, the adequacy of antegrade aortic flow to the upper body and the status of the stented ductus (ie, the ductal arch) are assessed preoperatively with noninvasive imaging and with cardiac catheterization. After resternotomy, CPB with mild hypothermia is initiated using dual arterial cannulation into the ascending aorta and pulmonary trunk. The bands are removed from the branch PAs and replaced with tourniquets. The ascending aorta is clamped and the heart arrested with cardioplegia. An incision is made into the pulmonary trunk and its arterial cannula is replaced with a balloon-tipped cannula advanced through the ductal arch for lower-body perfusion. Excess ductal stent material is excised. An incision is made into the right PA, and through this arteriotomy a catheter-based stent is deployed within the pulmonary trunk, bridging the right and left PA ostia (Figures 1, *B*, and 2). The stent is anchored in place with 2 6–0 Prolene sutures and then roofed over by suturing a pulmonary homograft baffle around the stent from ostium to ostium, leaving native PA tissue as the base of the baffle. The pulmonary trunk is closed primarily or with homograft patch augmentation to assure unobstructed lower-body flow over the baffle. The heart is deaired and the aortic clamp removed. A superior cavopulmonary connection is established (usually a BDG). Intraoperative dilation or restenting of the ductal arch under fluoroscopy may be carried out at this time if necessary. Early postoperative anticoagulation is recommended.

Patients are seen periodically because interstage dilation of the pulmonary and/or ductal stents may be necessary. Indications for the Fontan stage are similar to conventional indications. The Fontan is performed in the standard fashion, excepting that dual arterial cannulation is again necessary if CPB is employed.

## Patients

### Case 1

The patient was born full-term weighing 3.2 kg and with a diagnosis of trisomy 21 and unbalanced, right ventricle-dominant atrioventricular canal with a left ventricular volume index of 14 mL/m^2^. The left-sided outflow tract was unobstructed; however, the aortic arch and isthmus were hypoplastic. The child underwent a hybrid stage I procedure on day of life (DOL) 4 with insertion of a 7 × 20 mm Protégé stent (ev3/Medtronic, Minneapolis, Minn) into the ductus arteriosus. Cardiac catheterization at age 5 months demonstrated unobstructed flow into the ascending aorta with transverse arch hypoplasia and no appreciable flow through the isthmus. The mean left and right PA pressures distal to the bands were 27 mm Hg and 10 mm Hg, respectively.

The patient underwent HCS2 procedure in the hybrid operating room suite with intraoperative placement of Palmaz Genesis (PG) 2510B stent (Cordis, Bridgewater, NJ) into the previously stented ductal arch. An iCast 22 × 5 mm covered stent (Atrium Medical Corporation, Hudson, NH) was placed over an 8 mm balloon as the intrapulmonary baffle stent. The BDG was completed. Exit angiography demonstrated patency of the superior cavopulmonary anastomosis with flow into both branch PAs (Figure 2, *A*).

The patient did well initially and was extubated. On postoperative day (POD) 5, the patient developed hypoxemia with opacification of the left lung on chest radiograph. Echocardiography and computed tomographic angiography demonstrated no flow in the left PA. Heparin therapy was initiated. Catheterization confirmed extensive thrombus in the left PA and intrapulmonary baffle stent; transcatheter tissue plasminogen activator was administered. The patient required venovenous extracorporeal membrane oxygenation that was weaned on POD 22. Repeat angiography demonstrated unresolved thrombus in the stented and distal left PA. The patient died on POD 26 due to refractory hypoxemia.

### Case 2

**Figure 3:**
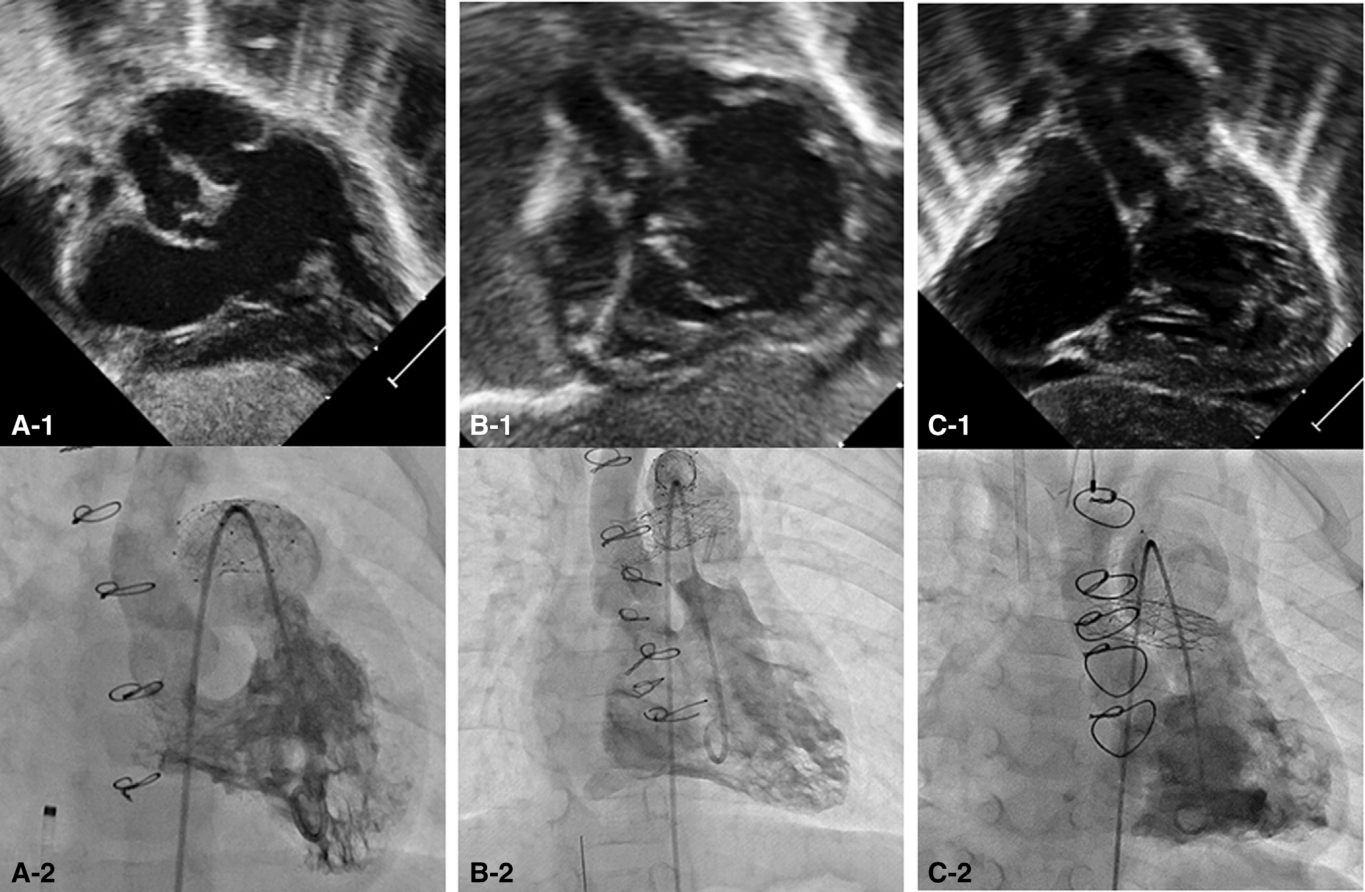
Initial subcostal echocardiograms (A-1, B-1, and C-1) and ventriculograms (A-2, B-2, and C-2) demonstrate the ventriculoarterial arrangements and relative sizes of the great vessel roots for patients 2 (double-outlet right ventricle), 3 (double-outlet right ventricle), and 4 (mitral atresia/ventricular septal defect), respectively. Adequacy of the native ascending aortic outflow is critical to demonstrate prior to attempt at the hybrid comprehensive stage II.

The patient was born full-term weighing 3.3 kg and with a diagnosis of double-outlet right ventricle with mitral atresia (Figure 3, *A*). The aortic root was located posteriorly with some subaortic crowding without obstruction; the distal aortic arch and isthmus were hypoplastic. The patient underwent hybrid stage I procedure on DOL 6 with stenting of the ductus with a 10 × 20 mm Protégé self-expanding stent. A balloon atrial septostomy was required on DOL 17. Interstage course was complicated by frequent respiratory infections requiring readmission. The patient required balloon dilation of the left PA band as well. Cardiac catheterization at 5 months showed acceptable pre-Glenn hemodynamic status with mean PA pressure 11 mm Hg bilaterally and no gradient from the ventricle to either the ascending or descending aorta.

The patient underwent HCS2 procedure with intraoperative placement of PG 1910B stent into the previously stented ductal arch. A second. PG 1910B stent was placed over an 8 mm balloon as the intrapulmonary baffle stent. The BDG was completed (Figure 2, *B*). The patient was extubated on POD 3 and managed postoperatively with heparin that was transitioned to enoxaparin before discharge; this was continued for 3 months before transition to aspirin. Between ages 11 and 33 months, the patient required a total of 4 catheterizations for recurrent ductal arch obstruction, including restenting of the ductal arch with an 8 × 26 mm Valeo stent (Bard, Tempe, Ariz) at 21 months and then with a PG 1910B stent expanded to 9 mm at 24 months. There was anatomic interruption of the aortic arch and descending aorta. Pre-Fontan catheterization at age 33 months showed acceptable pre-Fontan hemodynamic status with mean PA pressures 8 to 9 mm Hg; the ductal arch was balloon-dilated.

**Figure 4:**
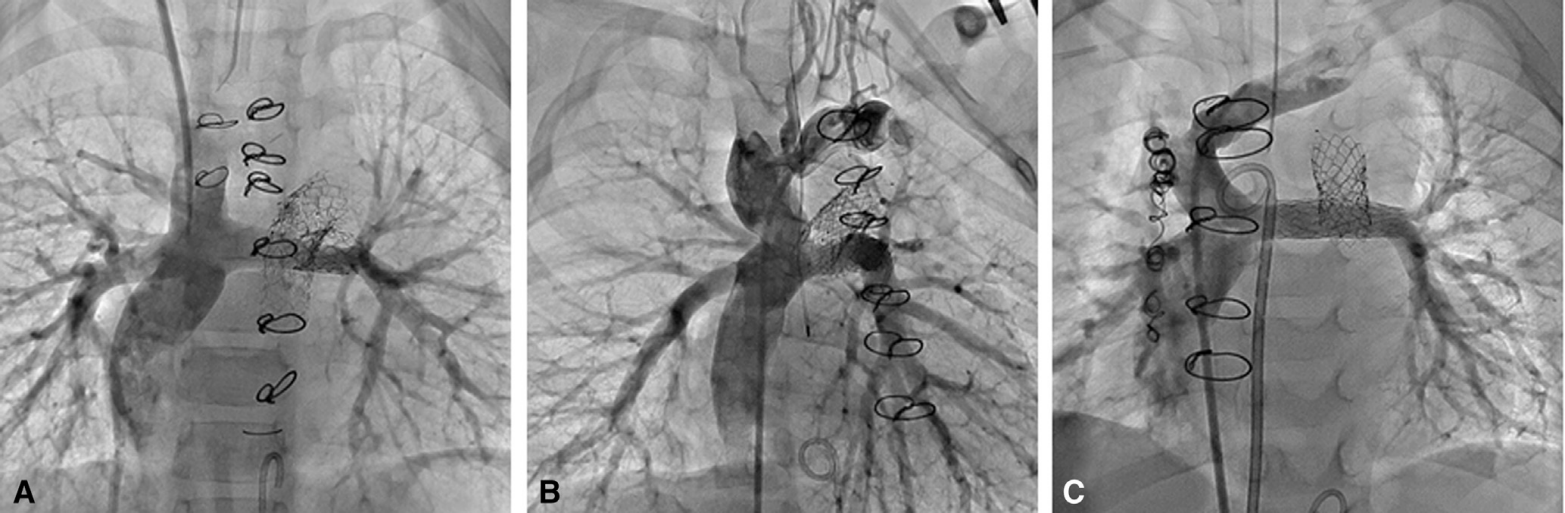
A, B, and C, Angiograms taken in patients 2, 3, and 4, respectively, after their Fontan completion surgeries demonstrate the cavopulmonary connections and long-term patency of the stented pulmonary artery baffles. Note the innominate vein stenosis in patient 3 (B) who had undergone banding followed by ligation of a left-sided superior vena cava.

At age 34 months, the patient underwent Fontan with an 18 mm extracardiac conduit and 4.8 mm fenestration (Figure 4, *A*). The postoperative course was complicated by respiratory infections. Sildenafil was initiated for fatigue and persistent cyanosis on follow-up. At age 4 years, the patient underwent cardiac catheterization at which point the left PA stent and ductal stents were redilated and the fenestration was closed. The patient was subsequently weaned off sildenafil and at most-recent follow-up at nearly age 6 years, the patient was asymptomatic with baseline oxygen saturation of 97% by pulse oximetry and with good exercise tolerance.

### Case 3

The patient was born full-term weighing 3.1 kg and with a diagnosis of double-outlet right ventricle with a parachute and hypoplastic mitral valve (Figure 3, *B*); diffuse transverse aortic arch hypoplasia was also present. The patient underwent hybrid stage I procedure on DOL 8 with stenting of the ductus with an 8 × 20 mm Protégé self-expanding stent. The left-sided superior vena cava was banded at this time in the presence of a small bridging innominate vein. A balloon atrial septostomy was required on DOL 17.

The patient did well during the interstage period. Cardiac catheterization at age 4 months showed acceptable pre-Glenn hemodynamic status with mean PA pressures 8 to 10 mm Hg and no significant gradient to the ascending aorta or across the ductal arch; the bridging vein was balloon-dilated due to stenosis. The patient subsequently underwent HSC2 with insertion of three stents to support the PA baffle; initially, a PG 2510B stent was inserted across the main PA and previously banded segments but this insufficiently covered the site of prior right PA banding. Therefore, an additional PG 1570PPS stent into the native right PA and an interposition PG 1270PPS stent, to bridge the 2 stents, were required. The right BDG was performed (Figure 2, *C*), the left-sided superior vena cava was ligated (as the innominate vein was of adequate size), and an atrial septectomy was performed. The postoperative course was uncomplicated and the patient was discharged on enoxaparin for a total of 6 weeks, which was then transitioned to aspirin.

The patient returned to the catheterization laboratory at age 10 months for balloon dilation of the left PA stent as well as 2 right PA branches due to stenosis. Cardiac catheterization at age 34 months showed appropriate pre-Fontan hemodynamic status with BDG pressure of 10 mm Hg, a trivial 5 mm Hg gradient to the native ascending aorta, and mild narrowing of the distal ductal arch stent with an 8 mm Hg gradient. There was anatomic interruption of the aortic arch and the descending aorta. At 37 months, the patient underwent Fontan with an 18 mm extracardiac conduit and 4.4 mm fenestration (Figure 4, *B*); an intraoperative PG 2910B stent was inserted into the ductal arch. He did well postoperatively with sildenafil started before discharge for moderate cyanosis with oxygen saturation mid-80%.

At age 45 months, cardiac catheterization showed Fontan pressures of 10 mm Hg; the ascending aorta, ductal arch, and left PA were unobstructed. The patient underwent balloon dilation of the innominate vein and the fenestration was closed. At most-recent follow-up at age 4 years, the patient was well and weaning off sildenafil; baseline oxygen saturation was 98% on room air and there was no report of exercise intolerance.

### Case 4

The patient was born full term weighing 3.0 kg with pregnancy complicated by maternal Zika virus infection. A cardiac diagnosis of mitral atresia with a moderate-to-large conoventricular septal defect was not made until DOL 9 upon evaluation for desaturation not responsive to supplemental oxygen. At the time of diagnosis, the ductus arteriosus was restrictive in the setting of transverse arch hypoplasia and coarctation of the aorta; right ventricle function was severely depressed. There was override of the native aorta with aneurysmal valve tissue near the ventricular septal defect, but this did not cause obstruction (Figure 3, *C*). The patient was also noted to have supraventricular tachycardia that responded to adenosine and did not recur. After initiation of prostaglandin E-1 as well as enteral digoxin, the ductus increased in size and right ventricle function gradually improved.

On DOL 20, the patient underwent delayed hybrid stage I procedure with stenting of the ductus with an 8 × 20 mm Protégé self-expanding stent. Postoperative course was complicated by delayed sternal wound healing and poor feeding requiring gastrostomy tube insertion. At age 5 months, the patient underwent catheterization that showed mean PA pressures 19 to 20 mm Hg distal to the PA bands; right ventricular end-diastolic pressure was 10 mm Hg. Cardiac magnetic resonance imaging was obtained that confirmed an adequately sized ventricular septal defect.

At age 9 months, due to some delays related to patient illnesses and social concerns, the patient underwent HCS2 procedure with insertion of a PG 2910B stent as the intrapulmonary baffle stent (Figure 2, *D*). The main PA and proximal ductal arch were augmented with pulmonary homograft. The patient did well postoperatively and was discharged home on anticoagulation with enoxaparin for 2 months before transitioning to aspirin. The patient required balloon dilation of the PA stent 2 months postoperatively due to proximal narrowing.

Pre-Fontan catheterization at age 2.5 years showed a BDG pressure of 14 mm Hg and right ventricular end-diastolic pressure 10 mm Hg; balloon dilation of the PA stent and the ductal arch stent were undertaken with resolution of mild gradients. The aortic arch and descending aorta remained in continuity, though high-grade stenosis remained at the native aortic isthmus. At age 3 years, the patient underwent Fontan with an 18 mm extracardiac conduit and 3.6 mm fenestration (Figure 4, *C*); an intraoperative PG 2910B stent was inserted into the ductal arch. Postoperative course was complicated by spontaneous fenestration closure due to thrombosis and recurrent pleural effusions. The patient required stenting of the fenestration on POD 14 and was discharged on sildenafil, enoxaparin, and diuretics. At most-recent follow-up at age 3.5 years, the patient is doing well, has transitioned from enoxaparin to aspirin, and is weaning from diuretics.

## Discussion

Four patients have undergone a novel approach to the stage II operation, applicable to a subset of patients with SV physiology having adequate antegrade aortic flow to the upper body. This HCS2 procedure offers advantages over the conventional comprehensive stage II operation insofar as arch reconstruction and creation of the Damus-Kaye-Stansel anastomosis are avoided, thus simplifying the operation and perfusion strategy. The HCS2 is currently completed through a limited PA incision and mild hypothermia. By avoiding mobilization and reconstruction of the distal arch, the risks of bleeding, recurrent laryngeal nerve injury, and phrenic nerve injury are decreased.

As with any new procedure, we have experienced a learning curve with this operation. Notably, the first patient died from a thrombotic complication originally attributed to the covered PA stent. This led to further avoidance of covered stent placement into the PA baffle and to early postoperative initiation of anticoagulation. PA thrombosis following SV palliation is a well-described phenomenon.^13^, ^14^, ^15^, ^16^ The patient, furthermore, was a high-risk patient with SV physiology given a history of trisomy 21 and known elevation in left PA pressure before HCS2 procedure. It is plausible that the patient's complication was related to these clinical factors and not necessarily to the covered stent. This possibility is important because safe use of a covered stent in lieu of a surgically placed intrapulmonary baffle could further simplify the HCS2 by allowing this stent to be placed without CPB. In this way, one can envision a patient with SV physiology undergoing all 3 palliative stages with a beating heart, and with warm CPB required only during the BDG and Fontan completion.

**Figure 5:**
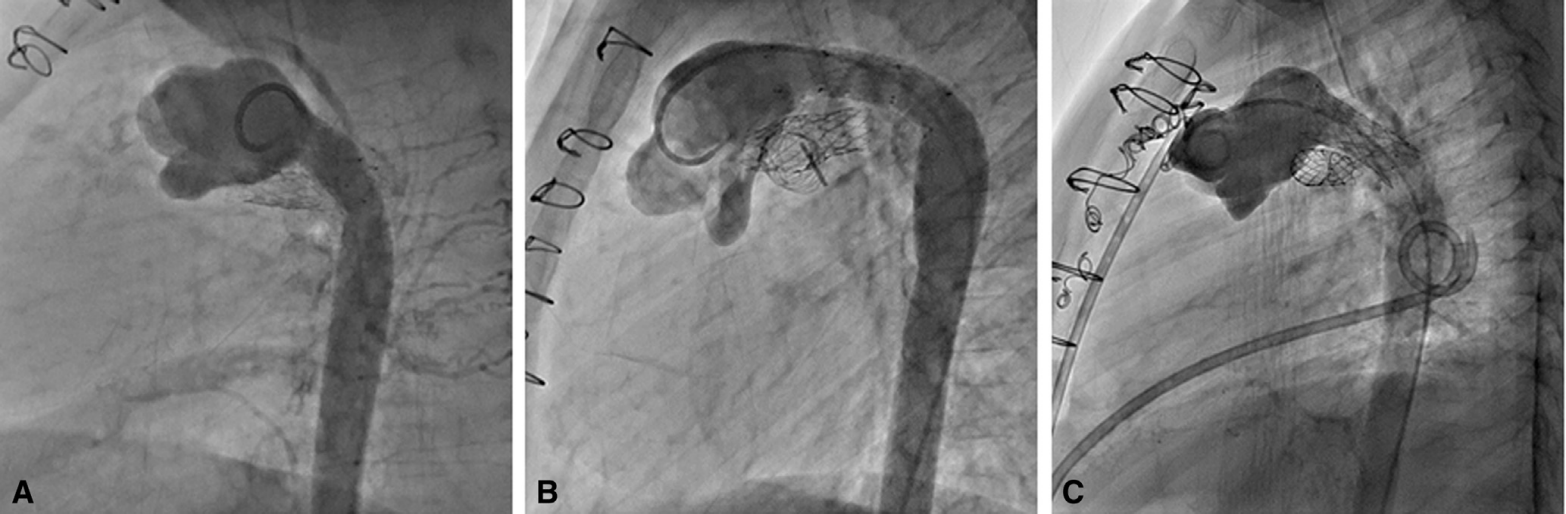
A, B, and C, Lateral angiograms taken with a pigtail catheter in the native main pulmonary artery demonstrate the ductal arches of patients 2, 3, and 4, respectively, which have been able to be expanded to support the somatic growth of the patient. Note the relative dilation of the native main pulmonary artery root after anterior patch augmentation, with the stented pulmonary artery baffle residing posteriorly. Also note the small amount of retrograde filling of the native distal aortic arch in (A) and (C).

The number of transcatheter interventions that have been required postoperatively to manage the PA baffle and ductal stents is of concern. All patients have required restenting of the ductal arch (Figure 5). Catheter-based interventions and stenting of arterial structures are not uncommon in patients undergoing the staged palliation for SV heart disease, especially if an initial hybrid strategy is employed.^17^^,^^18^ As our experience with the procedure grows, so does our ability to predict and plan for interventions with the goal of reducing the additional catheter-based procedures that have been required to date. This includes the now-routine use of intraoperative stenting of the ductal arch to avoid risk of femoral vessel injury in a percutaneous approach. One might also envision that, at the time of the initial stage I hybrid procedure, the use of a balloon-expandable stent with capacity to reach adult size would lessen the need for restenting of the ductal arch. Such stents are commonly used in catheter-based management of coarctation of the aorta, and show good resistance to in-stent stenosis in this disease.^19^ In addition, computational dynamics studies have shown that aortic segments stented for coarctation confer a relatively small impedance increase and thus do not substantially affect ventricular afterload.^20^ Late ductal arch obstruction remains a possibility following the HCS2, and if this cannot be managed by transcatheter techniques, then late surgical augmentation of the ductal arch would be required. Although our sample size is small, no patient has required surgical augmentation to date.

**Figure 6:**
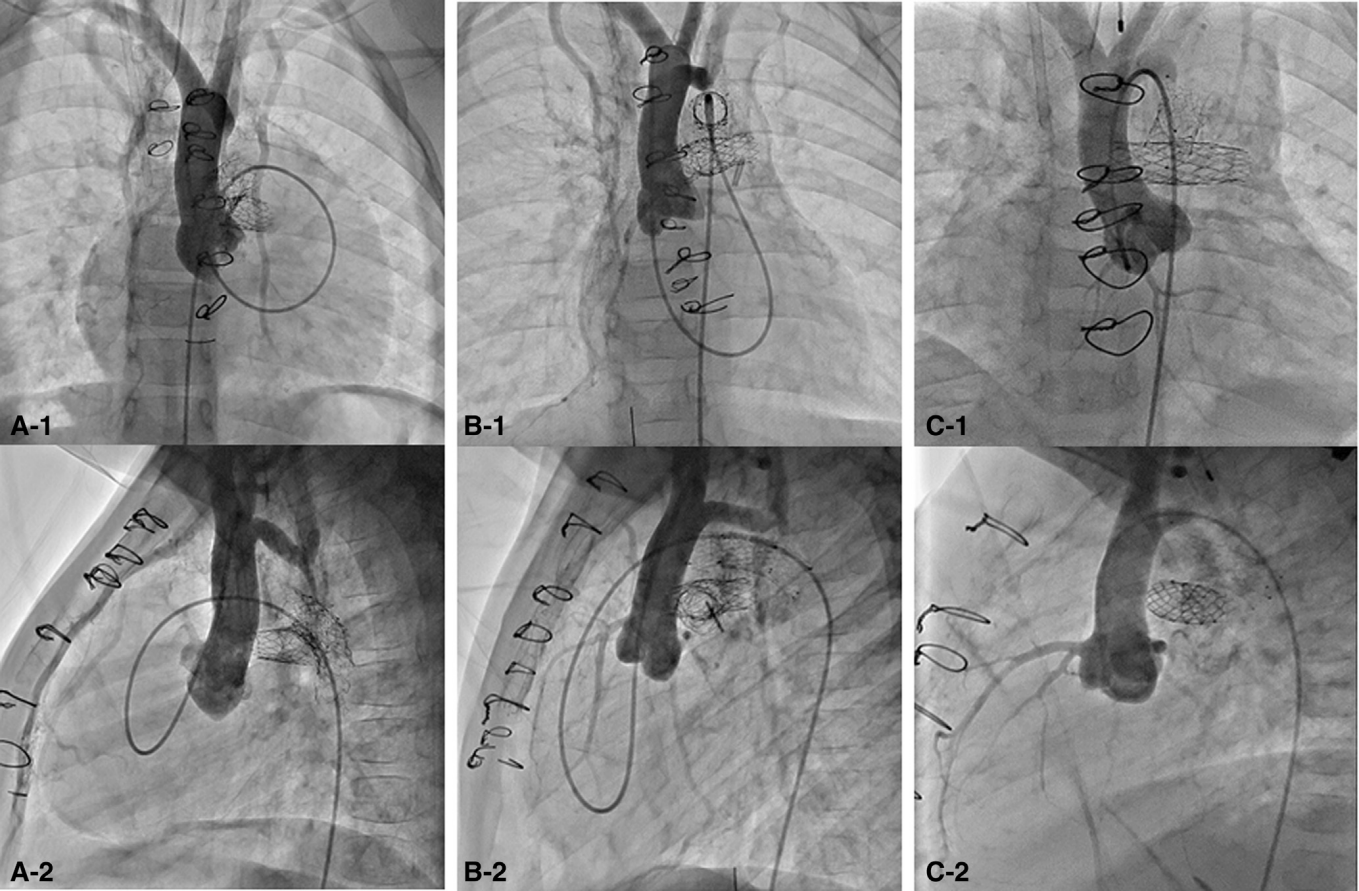
A-1, B-1, and C-1, Anterior-posterior and (A-2, B-2, and C-2) lateral projections of angiograms in the native ascending aorta in patients 2, 3, and 4, respectively, demonstrating the adequate growth of these vessels to supply circulation to the upper body. Note that in patients 2 and 3, due to functional or near interruption of the aortic arch, access to the native ascending aorta is obtained from a femoral arterial approach by crossing the native pulmonary valve via the ductal arch in a retrograde fashion, looping within the single ventricle, and then crossing the aortic valve in an antegrade fashion. In patient 4, direct retrograde access to the ascending aorta is obtained across a coarctation in the native aortic arch.

A curious phenomenon associated with the HCS2 is that the patients are left with functional or even anatomic interruption of the aortic arch with separation of the upper and lower body circulation (Figure 6). This is a unique physiologic situation of parallel systemic circulations, and the long-term clinical consequence of this is unknown. To date, none of our patients have experienced clinical sequelae attributable to this dual circulation (the oldest patient is currently aged 6 years). The native aortic valves and ascending aortae of the surviving patients have exhibited growth commensurate with their somatic growth, remaining free of outflow tract obstruction. In the future, exercise testing and cardiac magnetic resonance imaging in these patients may provide valuable insights into the physiology of this unique circulation.

Finally, the anatomy within the pulmonary trunk is also unique in this reconstruction. The stented baffle establishing continuity between the branch PAs traverses the posterior lumen of the lower body outflow tract (Figures 1, *B*, and 5). The resulting flow pattern is complex. The net stress on the baffle stent is inward, rather than outward, leading to the potential concern that pulsatile lower-body systemic blood flow can distort the stent. Initial computational fluid dynamics studies of this complex circulation by our group are reported in the accompanying paper published in this issue of the Journal.^21^

## Conclusions

The HCS2 is a feasible alternative to the conventional comprehensive stage II operation in a subset of patients with SV physiology with adequate native ascending aortic outflow. As expected, we have seen reduction in the operative complexity of the stage II procedure. However, there has been a tradeoff with need for multiple postoperative transcatheter interventions, largely related to maintaining patency of the ductal arch. We have developed strategies to try to minimize the number of catheter-based reinterventions required. We will follow long-term outcomes, both clinical and physiologic, closely. We believe that this approach can be safely considered in select patients with SV physiology to reduce the cumulative burden of operative trauma, which has been the primary goal of the hybrid approach.

### Conflict of Interest Statement

The authors reported no conflicts of interest.

The *Journal* policy requires editors and reviewers to disclose conflicts of interest and to decline handling or reviewing manuscripts for which they may have a conflict of interest. The editors and reviewers of this article have no conflicts of interest.
